# Sunitinib inhibits lymphatic endothelial cell functions and lymph node metastasis in a breast cancer model through inhibition of vascular endothelial growth factor receptor 3

**DOI:** 10.1186/bcr2903

**Published:** 2011-06-21

**Authors:** Yasuo Kodera, Yasufumi Katanasaka, Yuka Kitamura, Hitoshi Tsuda, Kazuto Nishio, Tomohide Tamura, Fumiaki Koizumi

**Affiliations:** 1 Shien-Lab, National Cancer Center Hospital, 5-1-1 Tsukiji, Chuo-ku, Tokyo 104-0045, Japan; 2 Department of Genome Biology, Kinki University Faculty of Medicine, 377-2 Ohno-higashi, Osaka-Sayama, Osaka 589-8511, Japan; 3 Division of Molecular Medicine, Graduate School of Pharmaceutical Sciences, University of Shizuoka, 52-1 Yada, Suruga-ku, Shizuoka 422-8526, Japan; 4 Department of Pathology and Clinical Laboratories, National Cancer Center Hospital, 5-1-1 Tsukiji, Chuo-ku, Tokyo 104-0045, Japan; 5 Department of Thoracic Oncology, National Cancer Center Hospital, 5-1-1 Tsukiji, Chuo-ku, Tokyo 104-0045, Japan

## Abstract

**Introduction:**

Metastasis is a common event and the main cause of death in cancer patients. Lymphangiogenesis refers to the formation of new lymphatic vessels and is thought to be involved in the development of metastasis. Sunitinib is a multi-kinase inhibitor that blocks receptor tyrosine kinase activity, including that of vascular endothelial growth factor receptors (VEGFRs). Although sunitinib is a clinically available angiogenesis inhibitor, its effects on lymphangiogenesis and lymph node metastasis remain unclear. The purpose of this study was to investigate the effects of sunitinib on vascular endothelial growth factor receptor 3 (VEGFR-3) and a related event, lymphangiogenesis.

**Methods:**

The effects of sunitinib on the degree of phosphorylation of VEGFR-2/3 and other signaling molecules was examined in lymphatic endothelial cells (LECs) treated with the drug; VEGF-induced LEC growth, migration, and tube formation were also examined. For the *in vivo *study, luciferase-expressing breast cancer cells were transplanted into mammary fat pads of mice; the microvessel and lymphatic vessel density was then measured after treatment with sunitinib and anti-VEGFR-2 antibody.

**Results:**

First, in human LECs, sunitinib blocked both VEGFR-2 and VEGFR-3 phosphorylation induced by VEGF-C or VEGF-D, and abrogated the activation of the downstream molecules extracellular signal-regulated kinase 1/2 (ERK1/2) and Akt. Furthermore, sunitinib attenuated the cell-proliferation activity induced by VEGF-C/D and prevented VEGF-C-induced migration and tube formation of the LECs; however, anti-VEGFR2 treatment shows only a partial effect on the growth and functions of the LECs. We used a breast cancer cell line expressing luciferase as a metastatic cancer model. Sunitinib treatment (40 mg/kg/day) inhibited the growth of the primary tumor transplanted in the mammary fat pad of the mice and significantly reduced the number of blood and lymphatic vessels in the tumor. Furthermore, the development of axillary lymph node metastasis, detected by bioluminescent imaging, was markedly suppressed. This effect of sunitinib was more potent than that of DC101, an anti-mouse VEGFR-2 antibody.

**Conclusions:**

The results suggest that sunitinib might be beneficial for the treatment of breast cancer by suppressing lymphangiogenesis and lymph node metastasis, through inhibition, particularly important, of VEGFR-3.

## Introduction

Metastasis is the main cause of therapeutic failure and death in cancer patients [1]. Tumor cells disseminate to distant organs through lymphatic vessels and blood vessels [2]. The status of metastasis to the regional lymph nodes is a prognostic factor in patients with malignancies and a determinant of the treatment course of patients [3]. Lymph nodes have also been proposed to serve as a cancer cell reservoir, providing a supportive environment for further movement of the cancer cells to distal organs [4,5]. Previously, this metastatic process was thought to be passively initiated via preexisting lymphatic vasculature; however, recent studies suggest that new lymphatic vessel formation, called lymphangiogenesis, actively contributes to lymphatic metastasis. In a clinical study, lymphatic vessel density in a tumor was correlated with the risk of lymph node metastasis and a poor prognosis [6]. Therefore, therapies targeting tumor lymphangiogenesis are expected to suppress the risk of development of metastasis and provide clinical benefit in cancer patients.

The lymphangiogenic process is regulated by numerous molecules, including the vascular endothelial growth factor (VEGF) and VEGF receptor (VEGFR) family. Among them, VEGF-C and VEGF-D are well-studied potent inducers of lymphangiogenesis [7]. A large number of clinical studies have demonstrated that the expression levels of VEGF-C/VEGF-D are markedly associated with lymphangiogenesis and lymph node metastasis in various types of cancers, including breast, ovarian, lung, and colon cancer [8]; their forced expression in cancer cells was shown to induce tumor lymphangiogenesis and lymph node metastasis in a preclinical model [9,10]. In *in vitro *analyses, VEGF-C and VEGF-D have been shown to induce various cellular functions of the lymphatic endothelial cells (LECs) that constitute lymphatic capillaries. These cellular functions include proliferation, migration, and tube formation, which are important for lymphatic vascular development [11,12]. Their receptors, VEGFR-2 and VEGFR-3, are tyrosine kinase receptors, and under VEGFC/D stimulation, undergo autophosphorylation and activate the downstream molecules Akt and extracellular signal-regulated kinase1/2 (ERK1/2) [13].

VEGFR-3 is expressed in all endothelial cells and is necessary for the formation of the primary vascular plexus in early development; subsequently, its expression becomes restricted, with the exception of the fenestrated blood vessels, to LECs [5]. The results of experimental studies indicate that VEGFR-3 signaling plays a key role in the development of the lymphatic vascular network [14,15]. VEGFR-2 is a well-known mediator of blood vessel formation and has been shown to be expressed in the LECs and lymphatic endothelium *in vivo *[16,17]. Furthermore, VEGFR-2 and VEGFR-3 form homodimer and heterodimer complexes in the LECs and exhibit cooperative and redundant functions in adult lymphangiogenesis [18].

Targeting of VEGF and VEGFR signaling in a tumor has been considered a therapeutic strategy. To achieve this, several approaches have been examined, including use of antibodies against VEGF receptors [19,20], soluble receptors [21], and small molecules [22-24]. In particular, multitargeting of small molecules has been reported to be highly effective in animal models. Given the complexity and redundancy of the VEGF signaling network, multitargeting may be a better strategy for effective inhibition of lymphangiogenesis.

Before this approach, angiogenesis inhibitors targeting the VEGF-A and VEGFR-2 signaling pathways were developed [25]. Sunitinib and bevacizumab are already approved angiogenesis inhibitors available for the treatment of renal cell carcinoma and metastatic gastrointestinal stromal tumors that prove resistant to imatinib. Sunitinib inhibits receptor tyrosine kinase activity, including that of VEGFR, PDGFR, KIT, FLT3, RET, and CSF1R. Although one of the main mechanisms underlying tumor growth inhibition by sunitinib appears to be its antiangiogenic activity exerted via its inhibitory action on VEGFR2 kinase activity in blood endothelial cells, its influence on lymphatic cell functions and tumor lymphangiogenesis remains unclear.

In this study, we evaluated the effect of sunitinib on lymphangiogenesis and lymph node metastasis. For this purpose, we used lymphatic dermal endothelial cells for *in vitro *analyses with the endogenous ligands VEGF-C and VEGF-D. To examine lymphangiogenesis *in vivo*, MDA-MB-231luc-D3H2LN (MDA-MB-231LN), a daughter cell line of the metastatic breast cancer MDA-MB-231, was used along with the bioluminescent imaging technology. We found that sunitinib inhibited VEGFR-2 and VEGFR-3 activity simultaneously, thereby inhibiting the cellular functions of the LECs, including proliferation, migration, and tube formation. In addition, it attenuated tumor lymphangiogenesis and lymph node metastasis in a mouse mammary fat pad (m.f.p.) model. This is the first article reporting that sunitinib, which is a clinically available angiogenesis inhibitor, blocks VEGFR-3 signaling in LECs to suppress lymphangiogenesis.

## Materials and methods

### Reagents and cells

Sunitinib (Symansis, Washdyke, New Zealand, and Pfizer, New York, NY) was resuspended in dimethyl sulfoxide (DMSO) and diluted in cell medium or PBS for the *in vitro *and *in vivo *assays. Recombinant human VEGF-C and VEGF-D were dissolved in sterile PBS containing 0.1% bovine serum albumin and stored at -80°C. The following antibodies were used: mouse monoclonal VEGFR-3 and phosphotyrosine (Upstate Biotechnology, Inc., Lake Placid, NY), ERK1/2, phospho-ERK1/2, Akt, phospho-Akt, c-Raf, phospho-c-Raf, MEK, phospho-MEK (Cell Signaling Technology, Beverly, MA), ras (abcam), CD31 (BD Pharmingen, San Jose, CA), lymphatic vessel endothelial hyaluronan receptor LYVE-1 (Upstate Biotechnology), and neutralizing VEGFR-2 antibody (R&D Systems, Minneapolis, MN). LECs were purchased from Lonza (Gaithersburg, MD) and maintained in EGM-2 according to the supplier's instructions. Cancer cell lines were purchased from the American Type Culture Collection (Manassas, VA). The MDA-MB-231luc-D3H2LN (MDA-MB-231LN) cell line was obtained from Caliper (Hopkinton, MA). An MDA-MB-231LN cell line stably expressing luciferase protein was established and maintained as described in [26].

### Immunoprecipitation and Western-blot analysis

Cells were cultured overnight in a serum-free medium. The medium was replaced with a medium containing DMSO or sunitinib for 2 hours, and the cells were then stimulated with VEGF-C/D for 10 minutes. Cells were rinsed with cold PBS and lysed with RIPA buffer (25 m*M *Tris HCl (pH 7.6), 150 m*M *NaCl, 1% NP-40, 1% sodium deoxycholate, 0.1% SDS, phosphatase inhibitor cocktail (Sigma, Saint Louis, MO), and complete protease inhibitor (Roche, Indianapolis, IN)). For detection of phosphorylated VEGFR-3, cell lysates were immunoprecipitated with anti-VEGFR-3 and then pulled down with protein G (Santa Cruz Biotechnology, Santa Cruz, CA). Samples were dissolved in SDS sample buffer and electrophoresed on 10% SDS-PAGE gel before being transferred to a PVDF membrane. To detect downstream signaling, confluent LEC cultures were stimulated and harvested as described. The same amounts of lysates were separated by 12% SDS-PAGE and then blotted onto a PVDF membrane. Phosphorylated proteins were detected with the respective antibodies, as described earlier. After detection, the antibodies were removed with stripping buffer (Pierce, Rockford, IL), and the membranes were reblotted with anti-ERK1/2 and anti-Akt.

### LEC proliferation assay

To evaluate the effect of inhibitors and antibodies on LEC growth, we used the MTS assay, as previously described [27]. In brief, a 200-μl volume of an LEC cell suspension was seeded into each well of a 96-well plate (3,000 cells/well). The cells were cultured overnight before the medium was replaced with a serum-free medium containing growth factors and inhibitors. The concentrations of VEGF-C and VEGF-D were 500 ng/ml. The cells were exposed to each drug at various concentrations and cultured at 37°C in a humidified atmosphere for 72 hours. After the culture period, 40 μl of MTS solution was added to each well, and the plates were incubated for a further 4 hours at 37°C. The growth-inhibitory effects of each drug were assessed spectrophotometrically (Spectra Max 190; Molecular Devices Corporation, Sunnyvale, CA). The results were evaluated with analysis of variance (ANOVA).

### LEC migration assay

LECs were suspended in a serum-free medium and seeded in the top chamber of a cell-culture insert (BD Biosciences, Bedford, MA) with DMSO or sunitinib. The inserts were placed in 24-well plates and incubated with medium containing VEGF-C for 24 hours. The migrated cells were fixed with glutaraldehyde and stained with crystal violet. The cell numbers were counted under a microscope, and relative migration was evaluated by dividing the number of sunitinib-treated cells by the number of control cells.

### Tube-formation assay

LECs were trypsinized and resuspended in a serum-free medium and then seeded on culture plates (2 × 10^4 ^cells/well). After overnight culture, collagen I gel (3 mg/ml) was layered on the plated cells containing DMSO, sunitinib, or anti-VEGFR2. Cells were incubated for 16 hours at 37°C, in a 5% CO_2 _atmosphere.

### Quantification of the secreted protein (ELISA)

Cancer cells were cultured in a 12-well plate (1.2 × 10^5^) with basal medium containing 0.1% bovine serum albumin for 24 hours. The amount of secreted VEGF-A, VEGF-C, and VEGF-D in the cultured medium was quantified by using ELISA detection kits (R&D Systems).

### The m.f.p. model

MDA-MB-231LN was trypsinized and resuspended in a 50% DPBS/50% matrigel. The cells were implanted into the left abdominal m.f.p. site of 6- to 7-week-old SCID mice. Five days after the transplantation, the mice were randomized and divided into the control and treatment groups. Depending on the group to which they belonged, the mice were orally administered either vehicle or sunitinib once a day for 2 weeks. Tumor volumes were measured with calipers every 3 or 4 days. After 2 weeks, the lymph node metastasis status was evaluated. All animal experiments were conducted with the approval of the Committee for Ethics in Animal Experimentation, and in accordance with the Guidelines for Animal Experiments of National Cancer Center.

### Immunohistochemical analysis of angiogenesis and lymphangiogenesis

After treatment, the tumors were excised and embedded in OCT compound. Six-micrometer-thick frozen sections were stained with antibody to CD31 (rat monoclonal antibody; BD Pharmingen) for blood vessels and antibody to LYVE-1 (rabbit polyclonal antibody; Upstate Biotechnology) for lymphatic vessels. Each type of vessel was visualized with EnVision Detection System (DAKO, Glostrup, Denmark) and counted as the number of structures per square millimeter. Three sections from five individual mice were evaluated and then statistically analyzed by using the Dunnett method.

### Evaluation of lymph node metastasis

Metastasis in the axillary lymph nodes was detected by using the IVIS Imaging System (Xenogen, Alameda, CA). At 10 to 15 minutes after luciferin injection, the mice were placed in the IVIS Imaging System and imaged in the ventral view. To confirm the presence of metastatic cancer cells, the lymph nodes and lungs were excised from the mice at necropsy and imaged *ex vivo*. Data were evaluated with the Dunnett multiple comparisons.

### Statistics

All data are presented as mean ± standard deviation. Results were considered statistically significant at *P *< 0.05.

## Results

### Sunitinib inhibited VEGFR-2 and VEGFR-3 signaling

**Figure 1:**
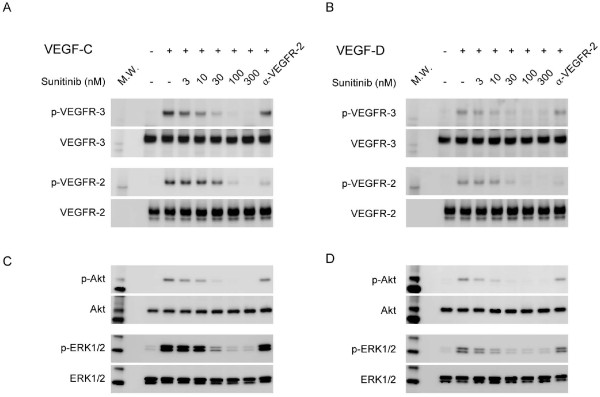
**Sunitinib blocked activation of VEGFR-2 and VEGFR-3 and their downstream molecules in human dermal LECs**. LECs were cultured to confluence, and the medium was replaced with a serum-free medium. After overnight incubation, the cells were pretreated with sunitinib, anti-VEGFR2 antibody, or DMSO (controls), before being stimulated with 200 ng/ml VEGF-C **(a) **or VEGF-D **(b)**. Lysates were immunoprecipitated with VEGFR-3-specific antibody for phospho-VEGFR-3 detection **(c, d)**. Other phosphoproteins were detected with their respective specific antibodies. All samples were separated by 4% to 20% gradient SDS-PAGE gel. The proteins were blotted onto a PVDF membrane and detected by Western blotting. Tyrosine phosphorylation of VEGFR-2 and VEGFR-3 was noted in the cells treated with VEGF-C or VEGF-D. Sunitinib blocked phosphorylation of both VEGFR-3 and VEGFR-2, whereas anti-VEGFR-2 antibody inhibited phosphorylation of only VEGFR-2. Increased phosphorylation of ERK1/2 and Akt was observed in the LECs stimulated with the VEGFs; however, this was attenuated in the cells treated with sunitinib. DMSO, dimethyl sulfoxide; LEC, lymphatic endothelial cell; MW, molecular weight marker; VEGF, vascular endothelial growth factor; VEGFR, vascular endothelial growth factor receptor.

To investigate the mechanism by which sunitinib inhibited lymphangiogenesis, we first used lymphatic vascular endothelial cells. Both VEGFR-2 and VEGFR-3 are expressed in LECs and are significantly phosphorylated by their natural ligands, VEGF-C and VEGF-D (Figure 1a, b). The receptor-activation potential of VEGF-C is higher as compared with that of VEGF-D. Sunitinib treatment suppressed these phosphorylations in a dose-dependent manner (IC_50 _= 48 n*M*). Sunitinib at similar concentrations has also been reported to inhibit VEGFR-3 in a cell-free kinase assay [25]. Taken together, sunitinib is actually an inhibitor of both VEGFR-2 and VEGFR-3 in the LECs. Phosphorylated VEGFRs are considered to mediate cellular functions by activating various types of signaling pathways. ERK1/2 and MEK, members of the mitogen-activated protein kinase family, are downstream molecules of VEGFR and play critical roles in cell growth. Protein kinase B (Akt) also mediates VEGFR signaling via phosphatidylinositol 3-kinase and regulates cell survival and proliferation. Even though ERK1/2, MEK, and Akt were significantly activated by VEGF-C/VEGF-D, sunitinib inhibited their phosphorylations (Figure 1c, d; Additional file 1 and 2). Because the expression levels of c-Raf and ras and the phosphorylation of c-Raf were not significantly affected by sunitinib, other Rafs or mediators could be involved in this pathway (see Additional data file 1).

To examine the contribution of VEGFR-2 in the stimulation of the VEGFs, we used an anti-VEGFR2 antibody that specifically inhibits VEGFR-2 phosphorylation (IC_50 _= 1.6 μg/ml) (see Additional data file 1, Figure S1C). When we treated the LECs with anti-VEGFR-2 antibody at 2 μg/ml, VEGFR-2 phosphorylation was reduced to almost the same level as that observed with sunitinib treatment at 100 n*M *(Figure 1). Interestingly, however, treatment with this antibody was not sufficient to abolish downstream signaling, although the downstream signaling molecules were strongly suppressed by sunitinib.

**Figure 2:**
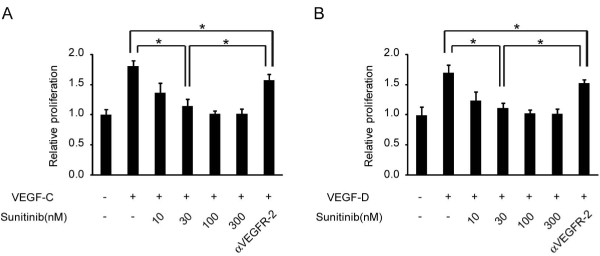
**Sunitinib inhibited VEGF-induced proliferation of LECs**. LECs were incubated in serum-free conditions in the absence or presence of 500 ng/ml of VEGF-C (left) or VEGF-D (right), and DMSO or 10 to 300 n*M *sunitinib. The cellular proliferation was quantified by MTS assay after 7 days of treatment. Columns indicate the values in six replicates normalized to the nontreated controls; bars, SD. *P < 0.05 compared with control. DMSO, dimethyl sulfoxide; LEC, lymphatic endothelial cell; VEGF, vascular endothelial growth factor; VEGFR, vascular endothelial growth factor receptor.

**Figure 3:**
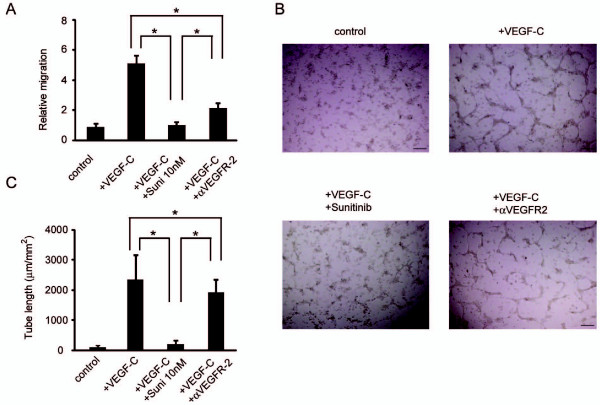
**Sunitinib inhibited VEGF-C-induced functions of LECs**. **(a) **Quantification of LEC migration induced by VEGF-C in cells treated with DMSO or 10 nmol/ml of sunitinib. Relative migration indicates the number of migrated cells normalized to the number of control cells. Columns indicate the values from four experiments; bars, SD. *P < 0.05 as compared with control. Sunitinib suppressed VEGF-C-induced LEC migration. **(b) **Photographs of VEGF-C-induced tube formation in cells treated with sunitinib or DMSO (control). VEGF-C promoted cord formation after overlay of type I collagen, which was suppressed by sunitinib treatment, but not by anti-VEGFR2 antibody. The black line represents 200 μm. **(c) **Quantification of tube formation. Columns indicate the values from the data of four fields; bars, SD. Sunitinib inhibited VEGF-C-induced tube formation. *P < 0.05 as compared with control. DMSO, dimethyl sulfoxide; LEC, lymphatic endothelial cell; Suni, sunitinib; VEGF, vascular endothelial growth factor; VEGFR, vascular endothelial growth factor receptor.

Sunitinib inhibited lymphatic endothelial cell growth and cell functions. To analyze the cellular functions, we first performed a growth-inhibition assay. Sunitinib inhibited the growth of LECs induced by VEGF-C and VEGF-D stimulation in a dose-dependent manner (Figure 2). At 100 n*M *of sunitinib, the growth of the LECs was completely inhibited. Conversely, anti-VEGFR-2 antibody (2 μg/ml) inhibited the cellular growth only partially. Migration assay and tube-formation assay were then performed. Transwell migration and capillary network formation of LECs were induced by VEGF-C, and sunitinib inhibited both functions at a lower concentration (10 n*M*) than that required for growth inhibition (Figure 3a). However, the inhibitory effects of anti-VEGFR-2 antibody treatment (2 μg/ml) on both migration and tube formation were weaker than those of sunitinib.

### Sunitinib inhibited tumor growth in the m.f.p. model

**Figure 4:**
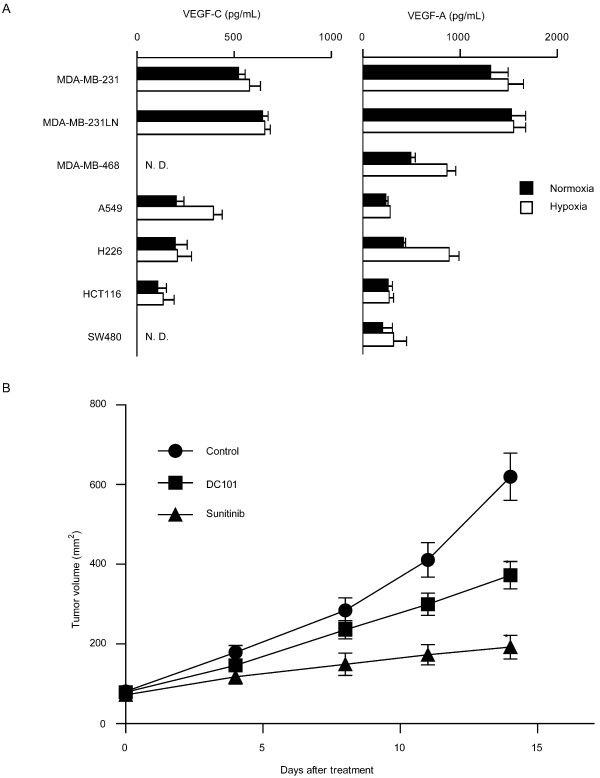
**Expression of VEGF-C in various cancer cell lines; sunitinib inhibited MDA-MB-231LN tumor growth**. **(a) **VEGF expression levels were quantified in metastatic cancer cell lines under normoxic and hypoxic conditions. All cells were cultured overnight under normoxic or hypoxic (5% O_2_) conditions; subsequently, the amounts of VEGFs secreted in the conditioned medium were quantified with enzyme-linked immunosorbent assay **(a)**. Columns indicate the values of four replicates; bars, SD. **(b) **Effects of sunitinib and of the anti-VEGFR-2 antibody, DC101, on the primary tumor growth. MDA-MB-231LN cells were transplanted into the m.f.p. of female mice, and the treatment was initiated on day 5 after the inoculation. Sunitinib was administered orally at 40 mg/kg once a day, and DC101 at 800 μg/mouse i.p. every 3 days for 2 weeks. Tumor volume was measured on the indicated days. Points indicate tumor volume; bars, SEM. *P < 0.05 as compared with control. m.f.p., mammary fat pad; N.D., not detected; VEGF, vascular endothelial growth factor; VEGFR, vascular endothelial growth factor receptor.

To examine the effect of sunitinib on lymphangiogenesis and metastasis, we used the mammary fat pad model with a highly metastatic breast cancer cell line MDA-MB-231LN. MDA-MB-231LN, which is engineered by transfection of the firefly luciferase gene into the MDA-MB-231 cell line, is a well-characterized model [26]. Before the animal experiments, we evaluated the expression of VEGFs in various types of metastatic cancer cell lines, including breast (MDA-MB-231, MDA-MB-468, MDA-MB-231LN), lung (A549, H226), and colon (HCT-116, SW480) cancer (Figure 4a). MDA-MB-231 and MDA-MB-231LN produce large amounts of VEGF-C and VEGF-A. In some cell lines, the expression of VEGF-A and VEGF-C was upregulated under hypoxic conditions. We also tried to detect VEGF-D expression; however, the amount of VEGF-D was below the limit of detection under both hypoxic and normoxic conditions.

Then we inoculated MDA-MB-231LN expressing luciferase into the m.f.p on the right side of the abdomen of a female mouse, and treated it with sunitinib at 40 mg/kg once a day, and DC101, an antibody for mouse VEGFR-2, at 800 μg/mouse for 2 consecutive weeks. In both groups of treated mice, tumor growth was significantly impeded (Figure 4b).

### Sunitinib inhibits lymphangiogenesis and lymph node metastasis

**Figure 5:**
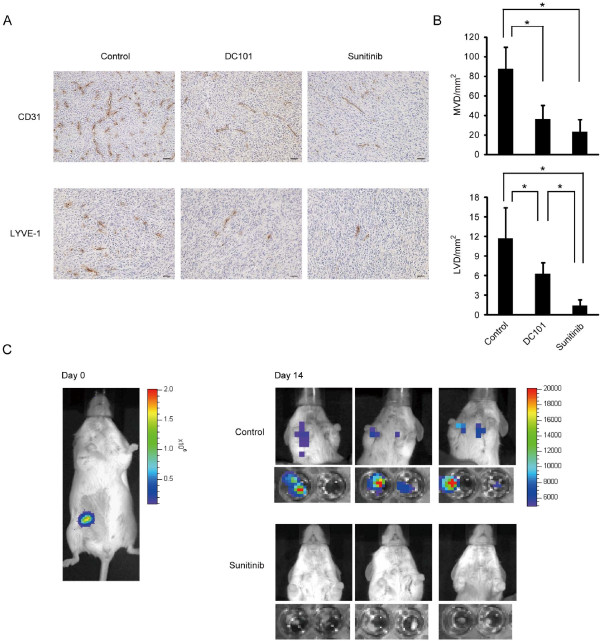
**Sunitinib inhibited angiogenesis, lymphangiogenesis, and lymph node metastasis in the m.f.p. model**. **(a) **Immunohistochemical analysis of the primary tumor treated with sunitinib, DC101, or vehicle. Tumors were stained with anti-CD31 for endothelial cells (upper) and anti-LYVE-1 for lymphatic endothelial cells (lower). Photographs were taken under a microscope at a magnification of ×8. Photographs of representative areas are shown. The black line represents 50 μm. **(b) **Effects of sunitinib and DC101 on tumor angiogenesis and lymphangiogenesis. The microvessel density (MVD, top) was determined by staining for CD31, and the lymphatic microvessel density (LVD, bottom) by staining for LYVE-1. *P < 0.05 as compared with control. **(c) **Lymph node metastasis was detected by bioluminescent imaging *in vivo*. The primary tumor (left) was identified on day 1 of treatment, and tumor metastasis around the axillary lymph node was detected after treatment with vehicle control (right, upper) or sunitinib (right, lower). *Ex vivo *data of the lymph nodes confirmed metastasis from the MDA-MB-231LN primary tumor. Three representative photographs from each treatment group are shown. LVD, lymphatic microvessel density; m.f.p., mammary fat pad; MVD, microvessel density.

Next, we examined the effect of sunitinib on angiogenesis and lymphangiogenesis by immunohistochemical analysis. The density of blood vessels stained with anti-CD31 in the primary tumor was significantly reduced, to almost the same degree, by treatment with sunitinib and DC101 (Figure 5a, b). The number of lymphatic vessels, stained with anti-LYVE-1, was also reduced by this treatment. Sunitinib was more potent than DC101 in reducing the number of lymphatic vessels (Figure 5a, b).

Then, the lymph node metastases were analyzed by bioluminescent imaging. Although previously, it was difficult to detect metastasis in living mice, development of luciferase and the bioluminescent technology has now enabled the detection of cancer cells in distant organs. Bioluminescent signals from the primary tumor in the m.f.p on the right side of the abdomen were observed on day 0 (Figure 5c, left panel). Signals from the cells metastasized around the axillary lymph node (right side) were detected in all the mice of the control group at day 14 (Figure 5c, right panels). Metastasis in the lymph node was confirmed by *ex vivo *imaging after resection. Lymph node metastasis on the opposite side (left side) was also observed in 6 mice from the control group; however, no metastases were recognized in the mandibular or mesenteric lymph nodes (data not shown). Metastasis to the axillary lymph node appears to occur in the early stage of the metastatic phase in this model.

Sunitinib strongly inhibited the development of axillary lymph node metastasis on the same side as the tumor (right side), although some of the mice showed metastasis (Table 1). Metastasis to the lung was also reduced (Table 1, Additional data file 3). DC101 tended to reduce metastasis; however, the effect was weak as compared with that of sunitinib.

**Table 1:** Effect of sunitinib and DC101 on axillary lymph node and lung metastasis from MDA-MB-231LN in the m.f.p. model

Treatment	Axillary lymph node metastases (right, left)	Lung metastases
Control	14/14 (13, 6)	12/14
DC101	10/15 (10, 1)	9/15
Sunitinib	5/15^a ^(5, 0)	3/15^a^

In the experiment illustrated in Figure 5, axillary lymph node and lung metastases were confirmed by bioluminescent imaging *ex vivo*. Values are shown as the number of mice with metastasis in each organ. The statistical analysis is described in the Materials and Methods section. The results were considered to be statistically significant at *P *< 0.05. ^a^*P *< 0.05 as compared with control.

## Discussion

Sunitinib is well-known as an inhibitor of angiogenesis and to exert activity against VEGFR2. In this study, we demonstrated that sunitinib inhibited the activation of VEGFR2/3 signaling and of the downstream molecules MEK, Erk, and Akt induced by VEGF-C and VEGF-D in lymphatic endothelial cells. Consequently, sunitinib blocked the cellular functions of the LECs, such as growth, migration and tube formation. Thus, sunitinib is actually a dual inhibitor of VEGFR2 and VEGFR3.

VEGFR-3 plays a pivotal role in lymphatic vascular formation, and VEGFR-3 inhibition by antibody has been shown to disrupt the cellular functions of LECs and adult lymphangiogenesis [18]. On the other hand, the role of VRGFR2 in the development of lymphatic vessels is not well clarified. However, VEGFR-2 is coexpressed and forms a heterodimer with VEGFR-3 in lymphatic endothelial cells [28] and the two molecules function together in inducing angiogenic sprouting [29]. Consistent with this mechanism, VEGFR2 and VEGFR3 in LECs are activated concurrently by VEGFC/D stimulation. In addition, anti-VEGFR2 antibody partially suppressed the functions of LECs and tumor lymphangiogenesis in the mouse model. VEGFR-2 could be involved in lymphangiogenesis through its direct effect on the LECs. Collectively, suppression of either VEGFR-2 or VEGFR-3 alone might not be sufficient to inhibit cellular signaling and lymphangiogenesis. Other multi-VEGFR inhibitors such as cediranib and PTK787 have also been demonstrated to show a suppressive effect on lymphangiogenesis [23,24]. Similarly, the dual inhibition of VEGFR-2 and VEGFR-3 by sunitinib might be important for the activity of this drug against the cellular functions of LECs and lymphangiogenesis.

Our results of the animal experiment imply that sunitinib might be of benefit in the treatment of breast cancer by suppressing lymph node metastasis. In clinical settings, lymph node metastasis is a useful marker of the prognosis of breast cancer and a determinant factor of systemic chemotherapy in a neoadjuvant setting; moreover, lymph node dissection prolongs the survival of patients with a positive sentinel lymph node. The main mechanism underlying this effect is thought to be suppression of the formation of lymphatic vessels as escape routes to neighboring lymph nodes. Furthermore, a new supportive role of lymph nodes for cancer cell survival in lymphatic organs has been proposed recently, and certain cancer cells seem to move toward distant sites via lymph nodes in the vicinity [5]. Given that the incidence of lung metastasis was also reduced by sunitinib treatment, restricting metastasis to regional lymph nodes might be useful to suppress systemic dissemination.

VEGFR pathways are attractive targets in cancer therapeutics, since the activities of the ligands and receptors can be controlled by specific antibodies or small-molecule inhibitors, including sunitinib. Also, the invasion-promoting role of VEGFRs in cancer cells has been reported recently [30]. Actually, numerous studies have shown that VEGFR inhibitors can suppress tumor growth and distant metastasis [31,32]. However, recently, two groups have also reported that short-term treatment with sunitinib promotes metastasis in mice [33,34]. These previous data and our present study data suggest that angiogenesis inhibitors and angiogenic therapy could affect tumor metastasis both positively and negatively depending on the drug dosing schedule or the therapeutic setting, although the precise underlying mechanisms remain unclear. Angiogenesis, lymphangiogenesis and metastasis are intricate processes involving not only numerous molecules, but also various physiologic responses. These mechanisms should be further investigated to develop better therapeutic use of inhibitors of angiogenesis and lymphangiogenesis.

## Conclusions

In this study, we demonstrated that sunitinib inhibited VEGFR-3 and VEGFR-2 signaling under VEGF-C or VEGF-D stimulation, and that it interfered with the cellular functions of LECs induced by VEGF-C, thereby inhibiting lymphangiogenesis and lymph node metastasis in a breast cancer model. In particular, we showed that inhibition of VEGFR-3 might be essential for these effects of sunitinib. Our findings suggest that sunitinib might be of benefit in the treatment of breast cancer by suppressing lymphangiogenesis and lymph node metastasis.

## Abbreviations

DMSO: dimethyl sulfoxide; ERK1/2: extracellular signal-regulated kinase 1/2; LEC: lymphatic endothelial cell; m.f.p: mammary fat pad; VEGF: vascular endothelial growth factor; VEGFR: vascular endothelial growth factor receptor.

## Competing interests

The authors declare that they have no competing interests.

## Authors' contributions

YKo is the first author of this article, conceived the study, organized it, and collected the data. YKa participated in the design of the immunologic assay. YKi collected some data related to the cellular assay. TH participated in the design of the immunohistochemical analysis and contributed to the analysis itself. KN participated in the design of this entire study and contributed to the interpretation of the results. TT participated in and contributed to the interpretation of the results. Fumiaki Koizumi participated in study design and coordination, helped draft the manuscript, and provided final approval for the version to be published. All the authors read and approved the final manuscript.

## Supplementary Material

Additional file 1 **Figure S1. effect of sunitinib on the signaling molecules ras, c-Raf, and MEK in the LECs, and the dose-dependent effect of anti-VEGFR-2 on VEGFR-2 phosphorylation**. Western blotting was performed, as shown in Figure 1. Although sunitinib did not affect the degree of phosphorylation of ras or c-Raf, it suppressed MEK phosphorylation **(a)**. LECs were treated with anti-VEGFR-2 (0, 0.2, 2. or 5 μg) under VEGF-C stimulation **(b)**. The inhibitory effect of the antibody was saturated at the antibody dose of 2 μg. Click here for file

Additional file 2 **Figure S2. Quantification of protein phosphorylations induced by VEGF-C/D**. Western blotting data (Figure 1) were captured and quantified by the imaging software, MultiGauge (Fujifilm). The amounts of phosphorylated protein were divided by those of total protein. Each phosphorylation ratio was normalized to that in the nontreated controls. Click here for file

Additional file 3 **Figure S3. Lung metastasis was detected after treatment with vehicle control (upper) or sunitinib (lower)**. Representative photographs of three *ex vivo *data from each treatment group are shown. Click here for file
