# Supplementary figures and images for: Sunitinib inhibits lymphatic endothelial cell functions and lymph node metastasis in a breast cancer model through inhibition of vascular endothelial growth factor receptor 3
Source: Breast Cancer Res. 2011 Jun 21;13(3):R66. doi: 10.1186/bcr2903 (PMC3218955; doi:10.1186/bcr2903)

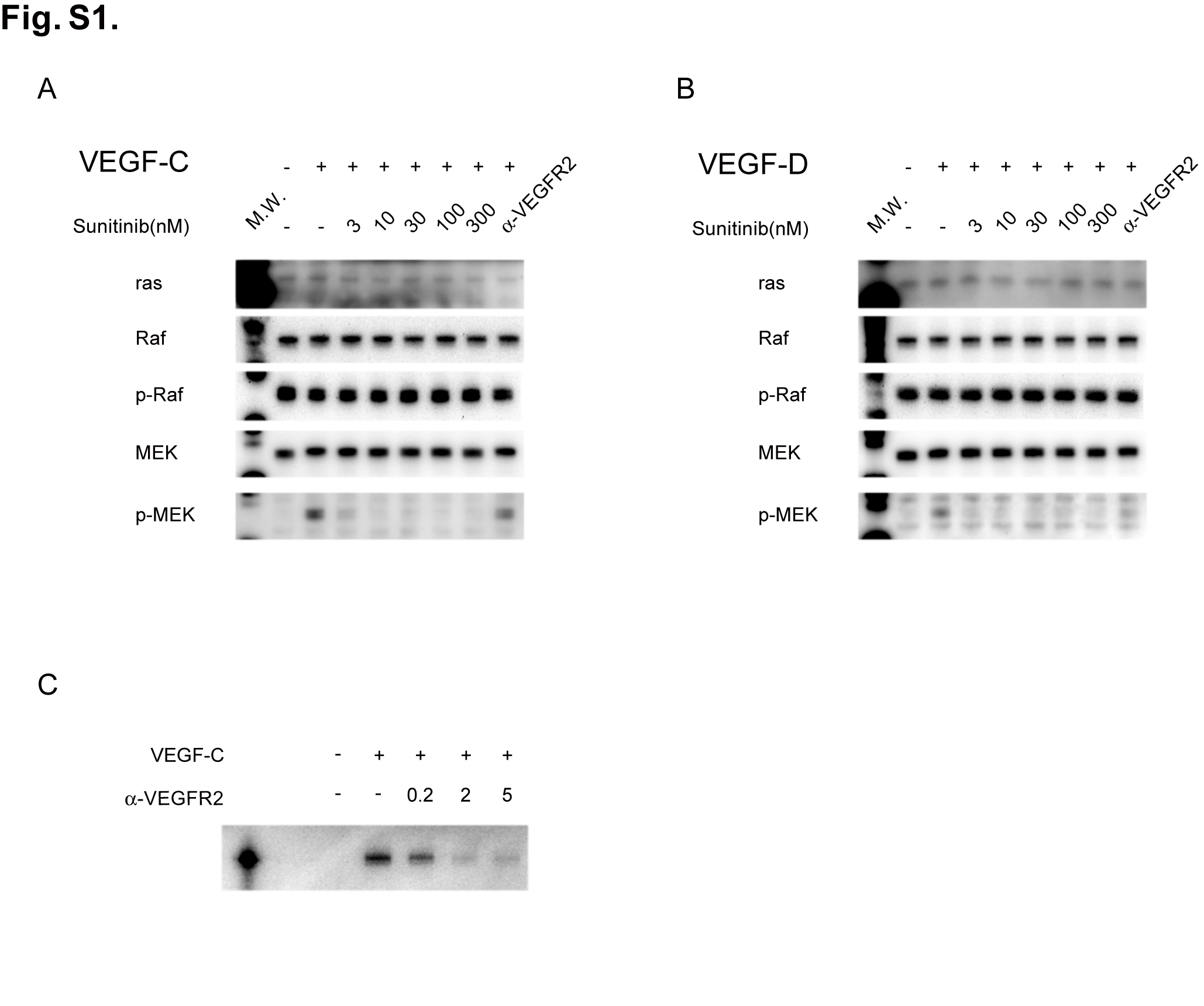

Supplement: Additional file 1 — Figure S1. effect of sunitinib on the signaling molecules ras, c-Raf, and MEK in the LECs, and the dose-dependent effect of anti-VEGFR-2 on VEGFR-2 phosphorylation. Western blotting was performed, as shown in Figure 1. Although sunitinib did not affect the degree of phosphorylation of ras or c-Raf, it suppressed MEK phosphorylation (a). LECs were treated with anti-VEGFR-2 (0, 0.2, 2. or 5 μg) under VEGF-C stimulation (b). The inhibitory effect of the antibody was saturated at the antibody dose of 2 μg. [file bcr2903-S1.TIFF]

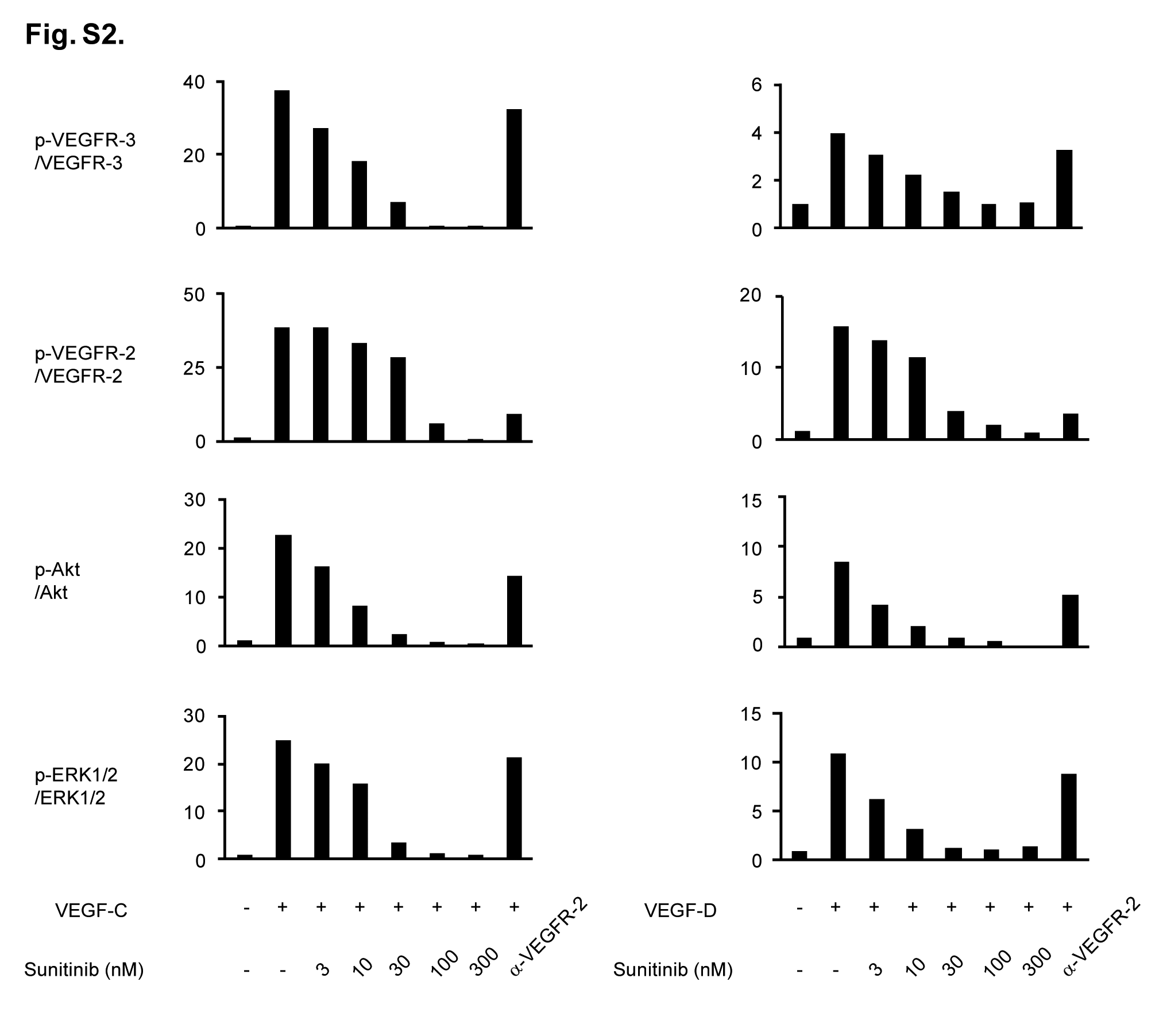

Supplement: Additional file 2 — Figure S2. Quantification of protein phosphorylations induced by VEGF-C/D. Western blotting data (Figure 1) were captured and quantified by the imaging software, MultiGauge (Fujifilm). The amounts of phosphorylated protein were divided by those of total protein. Each phosphorylation ratio was normalized to that in the nontreated controls. [file bcr2903-S2.TIFF]

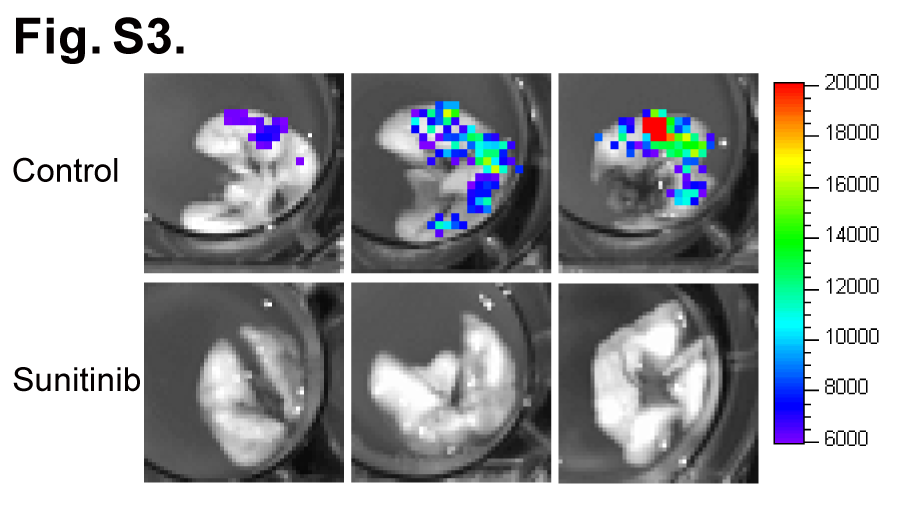

Supplement: Additional file 3 — Figure S3. Lung metastasis was detected after treatment with vehicle control (upper) or sunitinib (lower). Representative photographs of three ex vivo data from each treatment group are shown. [file bcr2903-S3.TIFF]
